# Porcupine inhibitors impair trabecular and cortical bone mass and strength in mice

**DOI:** 10.1530/JOE-18-0153

**Published:** 2018-05-02

**Authors:** Thomas Funck-Brentano, Karin H Nilsson, Robert Brommage, Petra Henning, Ulf H Lerner, Antti Koskela, Juha Tuukkanen, Martine Cohen-Solal, Sofia Movérare-Skrtic, Claes Ohlsson

**Affiliations:** 1 Centre for Bone and Arthritis ResearchInstitute of Medicine, Sahlgrenska Academy, University of Gothenburg, Gothenburg, Sweden; 2 Unit of Cancer Research and Translational MedicineMRC Oulu and Department of Anatomy and Cell Biology, University of Oulu, Oulu, Finland; 3 BIOSCAR UMRS 1132Université Paris Diderot, Sorbonne Paris Cité, INSERM, Paris, France

**Keywords:** Wnt signaling pathway, targeted therapy, osteoporosis, animal models

## Abstract

WNT signaling is involved in the tumorigenesis of various cancers and regulates bone homeostasis. Palmitoleoylation of WNTs by Porcupine is required for WNT activity. Porcupine inhibitors are under development for cancer therapy. As the possible side effects of Porcupine inhibitors on bone health are unknown, we determined their effects on bone mass and strength. Twelve-week-old C57BL/6N female mice were treated by the Porcupine inhibitors LGK974 (low dose = 3 mg/kg/day; high dose = 6 mg/kg/day) or Wnt-C59 (10 mg/kg/day) or vehicle for 3 weeks. Bone parameters were assessed by serum biomarkers, dual-energy X-ray absorptiometry, µCT and histomorphometry. Bone strength was measured by the 3-point bending test. The Porcupine inhibitors were well tolerated demonstrated by normal body weight. Both doses of LGK974 and Wnt-C59 reduced total body bone mineral density compared with vehicle treatment (*P* < 0.001). Cortical thickness of the femur shaft (*P* < 0.001) and trabecular bone volume fraction in the vertebral body (*P* < 0.001) were reduced by treatment with LGK974 or Wnt-C59. Porcupine inhibition reduced bone strength in the tibia (*P* < 0.05). The cortical bone loss was the result of impaired periosteal bone formation and increased endocortical bone resorption and the trabecular bone loss was caused by reduced trabecular bone formation and increased bone resorption. Porcupine inhibitors exert deleterious effects on bone mass and strength caused by a combination of reduced bone formation and increased bone resorption. We suggest that cancer targeted therapies using Porcupine inhibitors may increase the risk of fractures.

## Introduction

WNT ligands belong to a family of 19 secreted cysteine-rich glycoproteins that are essential for development and tissue homeostasis (Clevers & Nusse 2012). They signal through both the canonical WNT-β-catenin pathway and the noncanonical pathways (Moon *et al.* 2002, Kohn & Moon 2005). WNTs play crucial roles in the regulation of cell proliferation, survival, migration and polarity and self-renewal in stem cells. Abnormal WNT signaling in adults may contribute to diseases such as osteoporosis and cancer. The first demonstration of the link between WNT and cancer was that aberrant overexpression of WNT1 caused spontaneous mammary hyperplasia and retrovirus-induced mammary tumors in mice (Nusse & Varmus 1982). This finding was followed by further evidence of the role of WNTs in the tumorigenesis of various human cancers in β-catenin-dependent or -independent pathways (Anastas & Moon 2012). The importance of the WNT signaling pathway in bone homeostasis was highlighted by the identification of genetic variants in the WNT machinery that were responsible for rare diseases with either low or high bone mass (Baron & Kneissel 2013). Moreover, we previously identified WNT16 as a major contributor of cortical bone thickness and regulator of non-vertebral fractures risk (Zheng *et al.* 2012, Moverare-Skrtic *et al.* 2014). Finally, recent phase 3 studies have demonstrated that neutralizing sclerostin, a WNT antagonist mainly expressed by osteocytes, has a strong anabolic effect on bone mass and prevents osteoporotic fractures in post-menopausal women (Cosman *et al.* 2016, Saag *et al.* 2017).

Palmitoleoylation of WNTs by Porcupine is crucial for WNT trafficking from the endoplasmic reticulum to the membranous surface, for their secretion, and for their binding to their Frizzled receptor (Willert *et al.* 2003, Takada *et al.* 2006). NOTUM is a secreted lipase that selectively deacetylates WNTs by removing palmitoleate, thereby disrupting WNT signaling (Kakugawa *et al.* 2015, Nusse 2015). *Notum* gene deletion increases cortical bone mass in mice (Brommage *et al.* 2015). Mutations in the *PORCN* gene have been described in focal dermal hypoplasia (also called Goltz–Gorlin syndrome, OMIM Entry #305600). Patients with this disease display various skin manifestations and a wide range of skeletal abnormalities (Goltz 1992). Cases of concomitant giant cell tumors (Tanaka *et al.* 1990) and spontaneous fractures have been reported (Altschuler *et al.* 2012). Targeting Porcupine with pharmacological inhibitors to control WNT-dependent cancers is in clinical development, based on the previous demonstration of its efficacy in several pre-clinical studies, with apparent good tolerance (Liu *et al.* 2013, Proffitt *et al.* 2013, Boone *et al.* 2016, Madan *et al.* 2016). Among Porcupine inhibitors, LGK974 (also named WNT-974) is currently in a phase 1 study in patients with malignancies dependent on WNT ligands (ClinicalTrials.org - Nbib1351103). Wnt-C59 is another commonly used Porcupine inhibitor that has demonstrated its efficacy to disrupt the WNT signaling pathway in several cancer preclinical studies (Proffitt *et al.* 2013, Pradip *et al.* 2016).

However, in the context of WNT being a key regulator of bone mass, the effects of Porcupine inhibition on bone homeostasis are unknown. Global homozygous deletion of *Porcn* in mice leads to embryonic lethality, and the few heterozygotes that survive display major skeletal dysplasias, prohibiting proper characterization of adult bone phenotype (Barrott *et al.* 2011, Liu *et al.* 2012). Wntless (Wls) is a chaperone protein that specifically escorts WNT ligands during secretion, after palmitoylation by Porcupine. Osteoblast-specific deletion of Wls led to dramatic reductions of both trabecular and cortical bone mass and spontaneous fractures in mice (Zhong *et al.* 2012). Based on these findings, we hypothesized that inhibiting Porcupine could also lead to adverse effects on bone homeostasis. Thus, the aim of this study was to investigate the effects of pharmacological inhibitors of Porcupine on bone mass and strength in adult mice.

## Materials and methods

### Animals

Twelve-week-old female C57BL/6N mice (Charles River, Sulzfeld, Germany; *n* = 10 animals per group) were matched for body weight at baseline and treated by daily (except the two first weekends) oral gavage with either vehicle (DMSO, Merck), LGK974 at 3 mg/kg or 6 mg/kg (Selleckchem, Munich, Germany) or Wnt-C59 at 10 mg/kg (Selleckchem). All compounds were initially dissolved in pure DMSO, with subsequent dilutions step to obtain final solutions containing 5% DMSO, 1% carboxymethylcellulose (Merck) and 0.2% Tween 80 (Merck) for each treatment group. Mice received 17 doses of 100 µL of the drug solutions. Body weight was monitored weekly throughout the 21 days of the study. We replicated the findings in an independent experiment using vehicle and LGK974 at 4.5 mg/kg using a similar design. The latter experiment was used to collect blood samples from the tail vein at day 7.

All mice were housed in a standard animal facility under controlled temperature (22°C) and photoperiod (12 h of light, 12 h of darkness) and the animal care was in accordance with institutional guidelines with a normal diet. All animal experiments were approved by the Local Ethical Committee for Animal Research at the University of Gothenburg.

### Dual-energy X-ray absorptiometry

Analyses of total body and lumbar spine areal BMD (aBMD) were performed by dual-energy X-ray absorptiometry (DXA) using the Lunar PIXImus Mouse Densitometer (Wipro GE Healthcare, Madison, WI, USA) just prior to necropsy.

### High-resolution micro-CT

High-resolution micro-CT (μCT) analyses were performed on the lumbar vertebra L5 and femur using a Skyscan 1172 model micro-CT (Bruker micro-CT, Kontich, Belgium). The vertebra and femur were imaged with an X-ray tube voltage of 50 kV, a current of 200 μA, and with a 0.5-mm aluminum filter. The scanning angular rotation was 180°, and the angular increment was 0.70°. The voxel size was 4.49 µm isotropically. NRecon (version 1.6.9) was employed for image reconstructions. Trabecular bone in the vertebral body caudal of the pedicles was selected for analysis within a conforming volume of interest (cortical bone excluded) commencing at a distance of 4.5 µm caudal of the lower end of the pedicles, and extending a further longitudinal distance of 225 μm in the caudal direction. In the femur, the trabecular bone proximal to the distal growth plate was selected for analyses within a conforming volume of interest (cortical bone excluded) commencing at a distance of 786 μm from the growth plate and extending a further longitudinal distance of 135 μm in the distal direction. Cortical measurements were performed in the midshaft femur diaphyseal region of femur starting at a distance of 5.4 mm proximal from the distal growth plate and extending a further longitudinal distance of 135 μm in the distal direction.

### Mechanical strength

Before mechanical testing, tibias were rinsed in PBS. Three-point bending tests (span length, 4.5 mm; loading speed 0.155 mm/s) at the mid-tibia were made using an Instron 3366 Universal Testing Machine (Instron Corp., Norwood, MA, USA). Based on the recorded load deformation curves, the biomechanical parameters were calculated from raw files produced by Bluehill 2 software, version 2.6 (Instron) with custom-made Excel macros. 

### Bone histomorphometry

For the measurement of dynamic bone parameters, the mice were double labeled with alizarin and calcein (Merck), which were injected (intraperitoneally) into the mice 1 and 8 days before necropsy, respectively. Femur and L5 vertebrae were fixed in 4% paraformaldehyde, dehydrated in 70% ethanol and embedded in methyl-methacrylate. The femur was cut in half at the midshaft. The distal end was sectioned longitudinally to measure osteoclast numbers and surfaces at the midshaft cortical bone, in 4 μm thick sections stained in Masson–Goldner’s Trichrome, as previously described (Moverare-Skrtic *et al.* 2015). At the proximal end, the femur midshaft was sectioned in a transverse plane in an unstained 200 µm thick section to assess static and dynamic parameters. The L5 vertebra bodies were analyzed in 5  μm sections, and TRAP staining was performed to quantify the number of active osteoclasts, as described previously (Haÿ *et al.* 2009). Unstained sections (8 μm thick) were used to assess dynamic parameters. 

All parameters were measured using OsteoMeasure histomorphometry software (OsteoMetrics, Decatur, GA, USA) following the guidelines of the American Society for Bone and Mineral Research (Dempster *et al.* 2013). Femurs were analyzed by PharmaTest (Turku, Finland) and vertebras by Bioscar INSERM U1132 (Paris, France). Both laboratories were blinded to the treatment attributions.

### Serum biomarkers

Blood samples were collected at day 7 and at day 21 (necropsy) in two independent experiments. As a marker of bone resorption, serum levels of C-terminal type I collagen fragments were assessed using an ELISA RatLaps kit (CTX, Immunodiagostic Systems, Copenhagen, Denmark). Serum levels of amino pro-peptide of type 1 collagen (P1NP, Immunodiagostic Systems) were analyzed as a marker of bone formation.

### Primary bone cell cultures

Primary murine osteoblasts and osteoclasts were cultured in complete α-MEM medium (MEM alpha medium (Gibco) supplemented with 10% heat-inactivated FBS (Merck, F7524), 2 mM GlutaMAX (Gibco), 50 μg/mL gentamicin (Gibco), 100 U/mL penicillin and 100 μg/mL streptomycin (Gibco)).

Primary calvarial osteoblasts were isolated from newborn C57BL/6 mouse calvaria as described previously (Moverare-Skrtic *et al.* 2014). Cells were cultured for 3–4 days in complete α-MEM before subculture in 48-well plates at 20,000 cells/cm^2^ in complete α-MEM supplemented with 10 mM β-glycerophosphate disodium salt hydrate (BGP; Merck, G9422) and 0.2 mM L-ascorbic acid 2-phosphate sesquimagnesium salt hydrate (Asc-2P; Merck, A8960) with or without 100 ng/mL BMP-2 (R&D systems, Abingdon, UK; 355-BEC).

Mouse bone marrow macrophages (BMMs) were obtained as described previously (Takeshita *et al.* 2000, Granholm *et al.* 2007). For osteoclast generation, 20,000 BMMs were spot seeded in 48-well plates and cultured in complete α-MEM supplemented with 30 ng/mL M-CSF (R&D systems, 416-ML) with or without 4 ng/mL RANKL (R&D systems, 462-TEC).

For gene expression analysis, cells were lysed in RTL buffer (Qiagen, Sollentuna, Sweden) at indicated time points, followed by RNA purification, cDNA synthesis and real-time PCR as described below.

### Quantitative real-time PCR analysis

The harvested diaphysis of the tibia was flushed to remove bone marrow. Total RNA was prepared from diaphyseal cortical bone of the tibia, the vertebral body of vertebrae L6 and L3 (trabecular bone with bone marrow) and from soft tissues including liver, retroperitoneal and gonadal fat using TRIzol reagent (Life Technologies) followed by the RNeasy Mini Kit (Qiagen). The RNA was reverse transcribed into cDNA using High-Capacity cDNA Reverse Transcription Kit (#4368814, Applied Biosystems), and real-time PCR analysis was performed using predesigned real-time PCR assays (primers from Applied Biosystems) for Porcupine (*Porcn*, Mm00450403_m1), alkaline phosphatase (*Alpl*, Mm00475834_m1), cathepsin K (*Ctsk*, Mm00484036_m1), *Acp5* (encoding TRAP; Mm00475698_m1), osteoprotegerin (*Opg*, *Tnfrsf11b*, Mm00435452_m1) and rank-ligand (*Rankl*, *Tnfrsf11*, Mm00441908-m1) on the StepOnePlus Real-Time PCR system (Applied Biosystems). The mRNA abundance of each gene was adjusted for the expression of *18S* mRNA (4310893E).

### Statistical analysis

For statistical evaluation, we performed one-way ANOVA test followed by Dunnett’s test for multiple comparison, comparing all active treatments to the vehicle control group. For the *in vitro* time course expression studies, we used Tukey’s test for multiple comparison. For the mechanistic analyses (serum biomarkers, histomorphometry and gene relative expression), we only compared mice treated with LGK974 at 6 mg/kg to those treated with vehicle. In that setting, we used the Student’s *T*-test to compare the two groups. Results are presented as means with 95% CIs to the mean. All statistical tests were two sided, and the minimal level of statistical significance was *P* less than 0.05.

## Results

### Porcupine expression

**Figure 1:**
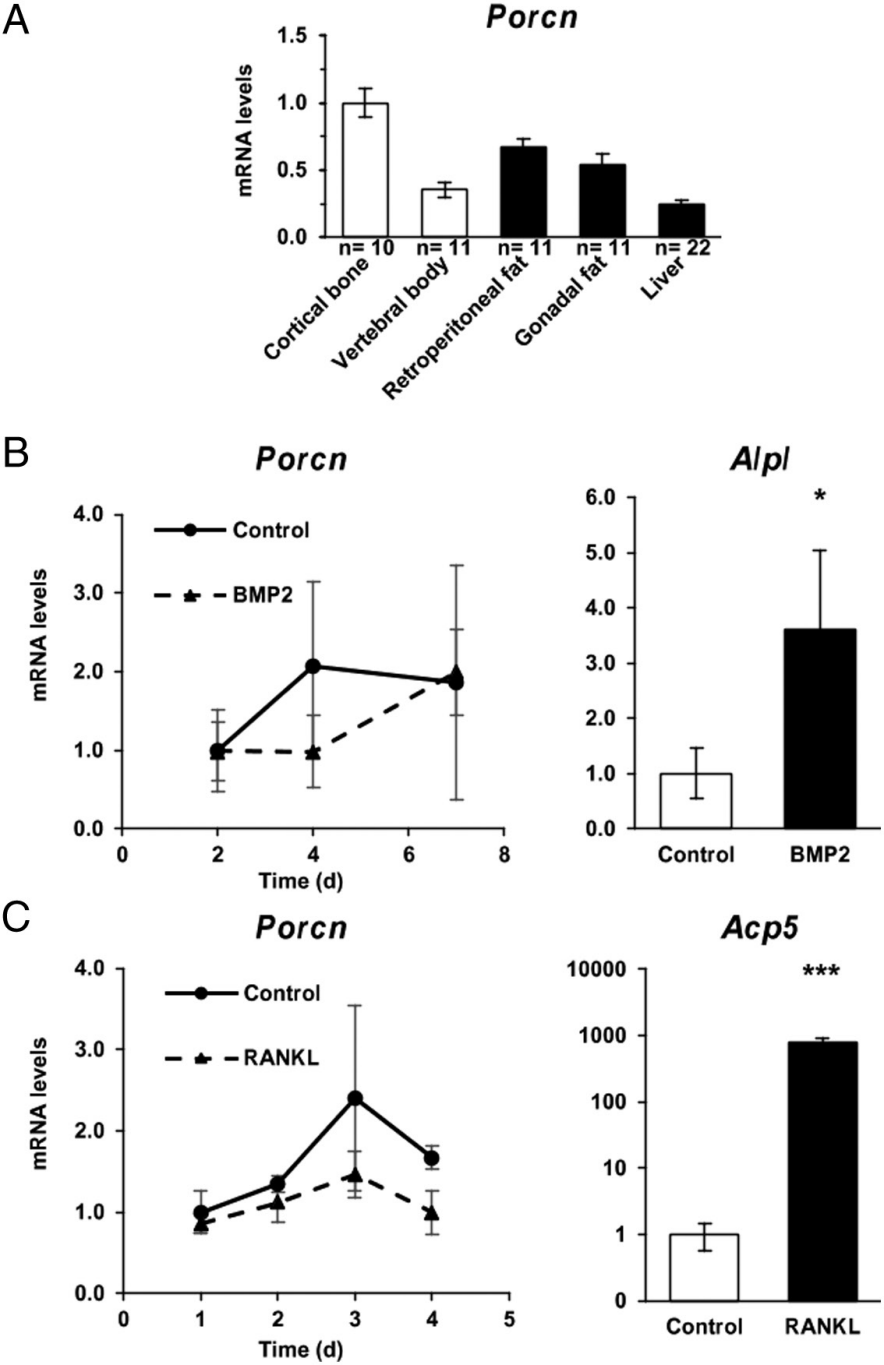
Porcupine expression. (A)* Porcn* mRNA levels in different tissues from n mice. (B) *Porcn* mRNA levels in calvarial osteoblasts cultured with or without BMP2 for 7 days (*n* = 4 wells/condition and time point). Results are expressed as fold-change to the levels in control osteoblasts at day 2. As a positive control, we show *Alpl* mRNA levels at day 7. (C) *Porcn* mRNA levels in bone marrow cell-derived osteoclasts cultured with or without RANKL for 4 days (*n* = 4 wells/condition and time point). Results are expressed as fold-change to the levels in control osteoclasts at day 1. As a positive control, we show *Acp5* mRNA levels at day 4. Bars represent the mean and error bars represent the 95% CI of the mean. *P* values are indicated as *P < 0.05; **P < 0.01; ***P < 0.001.

*Porcn* expression was compared in cortical diaphyseal bone, vertebral body (enriched in trabecular bone), fat and liver (Fig. 1A). *Porcn* was expressed in all tissues, but the mRNA levels were higher in cortical bone compared to vertebral body. In primary bone cell cultures, *Porcn* was expressed in both osteoblasts and osteoclasts. Osteogenic differentiation using BMP-2 increased *Alpl* expression as expected (*P* = 0.015), but did not alter *Porcn* expression in primary calvarial osteoblasts (Fig. 1B). Similarly, osteoclastic differentiation from BMMs by RANKL markedly increased *Acp5* expression (*P* < 0.001), but *Porcn* expression was unchanged (Fig. 1C).

### Total bone mineral density and bone turnover

**Figure 2:**
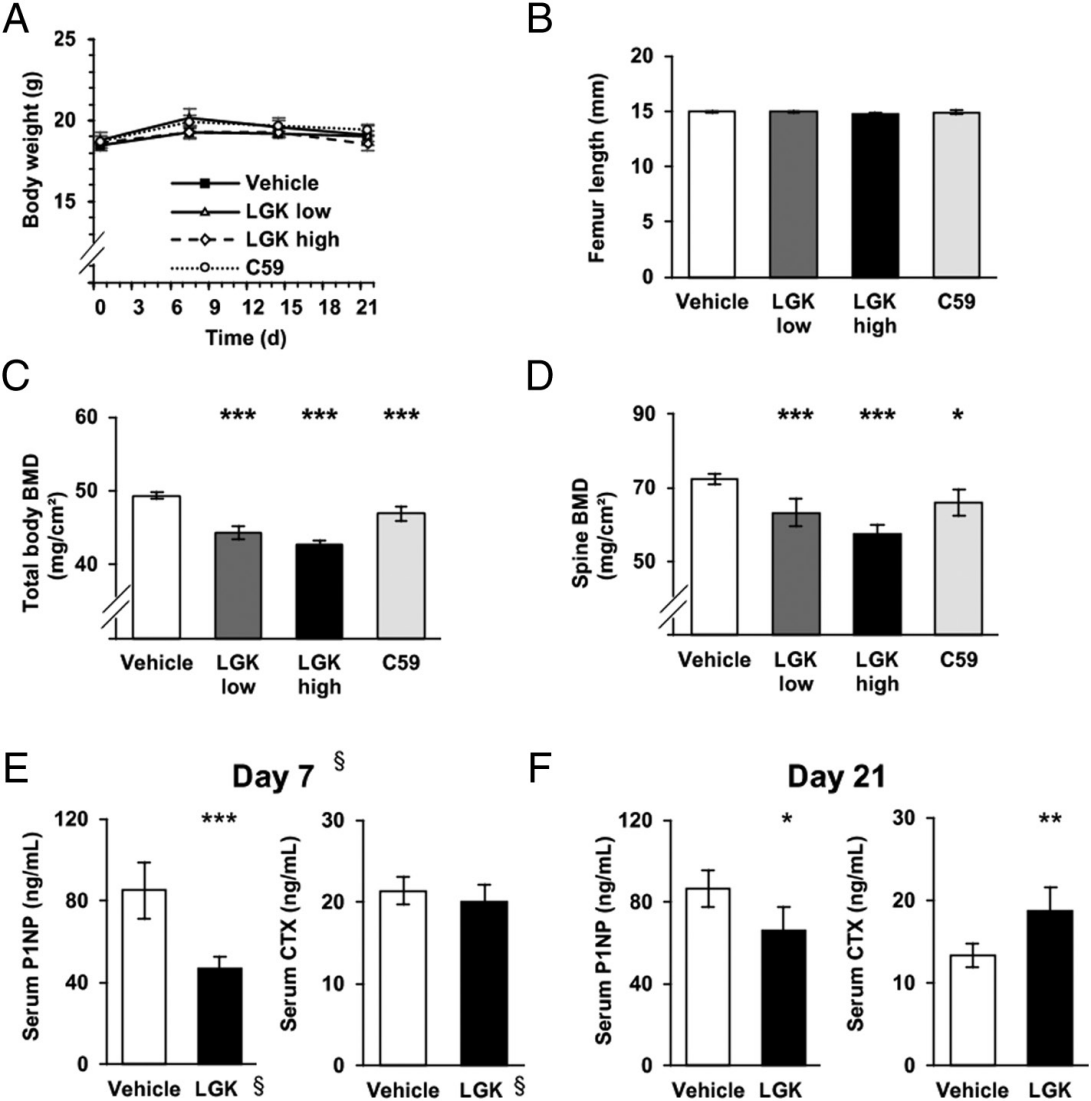
Systemic effects of Porcupine inhibitors. (A) Body weight changes over the study by treatment group (LGK low: LGK974 at 3 mg/kg/day; LGK high: LGK974 at 6 mg/kg/kg/day; C59: Wnt-C59). (B) Femur length at necropsy. (C) Total body bone mineral density (BMD) at necropsy. (D) Spine BMD at necropsy. (G) Serum levels of amino pro-peptide of type 1 collagen (P1NP) and C-terminal type I collagen fragments (CTX) at day 7. ^§^These results are obtained from a separate experiment with vehicle and LGK974 at 4.5 mg/kg/day. (E) Serum levels of P1NP and CTX at necropsy in animals treated with vehicle or LKG974 at 6 mg/kg/day. For each graph, *n* = 10 animals per group. Bars represent the mean and error bars represent the 95% CI of the mean. *P* values are indicated as *P < 0.05; **P < 0.01; ***P < 0.001.

Both Porcupine inhibitors were well tolerated and the treated mice displayed no difference in body weight or longitudinal bone growth assessed by femur length at the time of necropsy (Fig. 2A and B). Total body BMD (all treatments *P* < 0.001 vs vehicle, Fig. 2C) and spine BMD (Fig. 2D), assessed by DXA, were reduced by both Porcupine inhibitors. Both doses of LGK974 reduced BMD to the same extent.

For a better understanding of the mechanisms of action of Porcupine inhibitors in bone, we evaluated the effect of LGK974 on serum bone turnover markers at day 7 and day 21. LGK974 treatment substantially reduced the serum levels of the bone formation marker P1NP compared with vehicle treatment already at day 7 (Vehicle, 85.2 ng/mL, 95% CI 69.5–100.8; LGK974, 46.7 ng/mL, 95% CI 39.7–53.6; *P* < 0.001, separate experiment, Fig. 2E), and this effect was also significant at day 21 (*P* = 0.014; Fig. 2F). LGK974 increased the serum levels of the bone resorption marker CTX at day 21 (*P* = 0.004; Fig. 2F) while no significant effect was observed at day 7 (*P* = 0.349, Fig. 2E).

### Cortical bone impairment by Porcupine inhibitors

**Figure 3:**
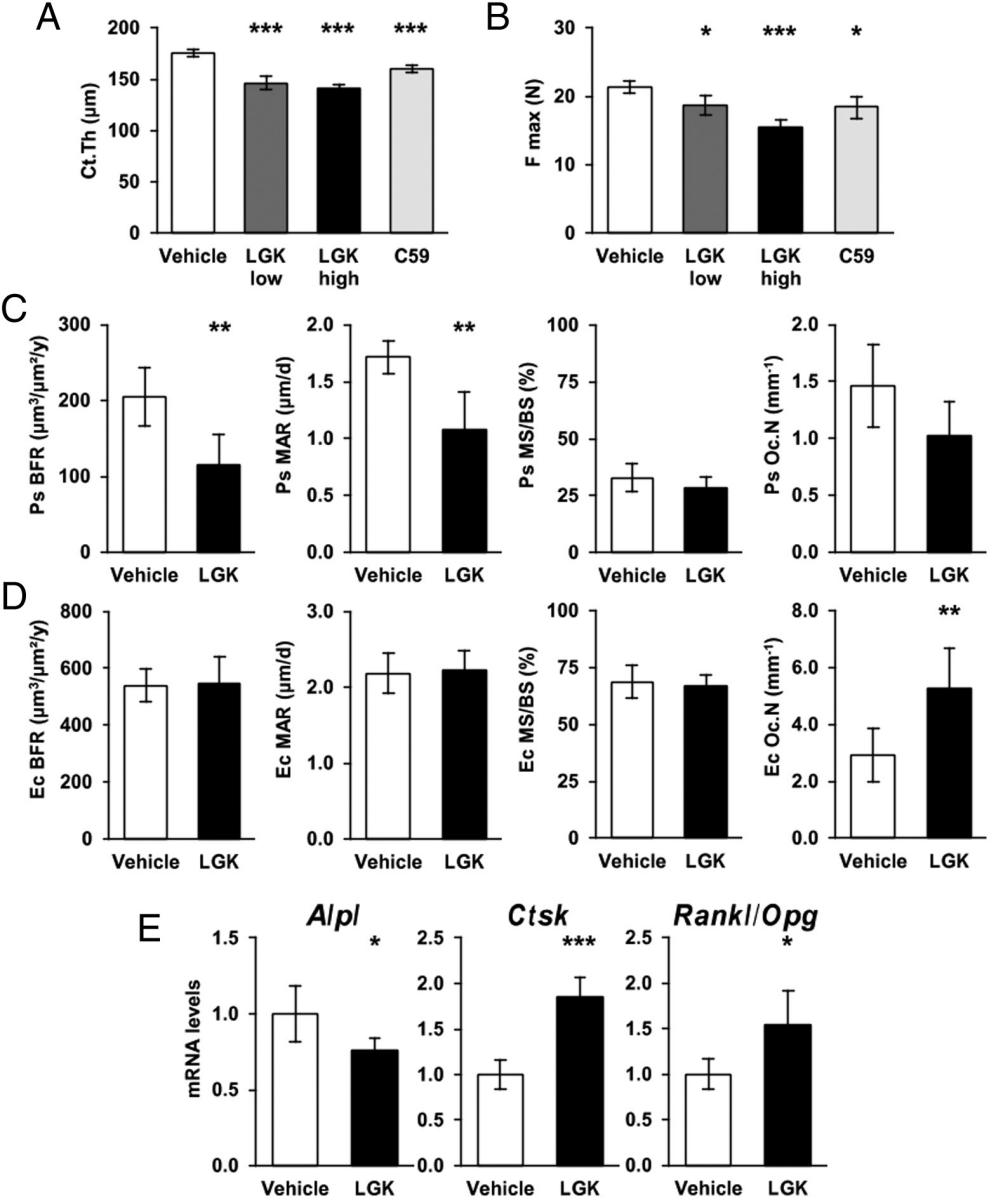
Effects of Porcupine inhibition on cortical bone. (A) Cortical thickness (Ct.Th) of the femur assessed by µCT. (B) Maximal Failure load (F max) of the tibia from the 3-point bending test. (C) Bone histomorphometry at the periosteal surface of femur cortical bone showing periosteal bone formation rate (Ps BFR), mineralization apposition rate (Ps MAR), mineralizing surface over bone surface (Ps MS/BS) and osteoclast number per bone perimeter (Ps Oc.N). (D) Bone histomorphometry at the endocortical surface of femur showing endocortical bone formation rate (Ec BFR), mineralization apposition rate (Ec MAR), mineralising surface over bone surface (Ec MS/BS) and osteoclast number per bone perimeter (Ec Oc.N). (E) *Alpl*, *Ctsk* and* Rankl/Opg ratio* mRNA levels in cortical bone. For each graph, *n* = 10 animals per group. Bars represent the mean and error bars represent the 95% CI of the mean. *P* values are indicated as *P < 0.05; **P < 0.01; ***P < 0.001.

**Figure 4:**
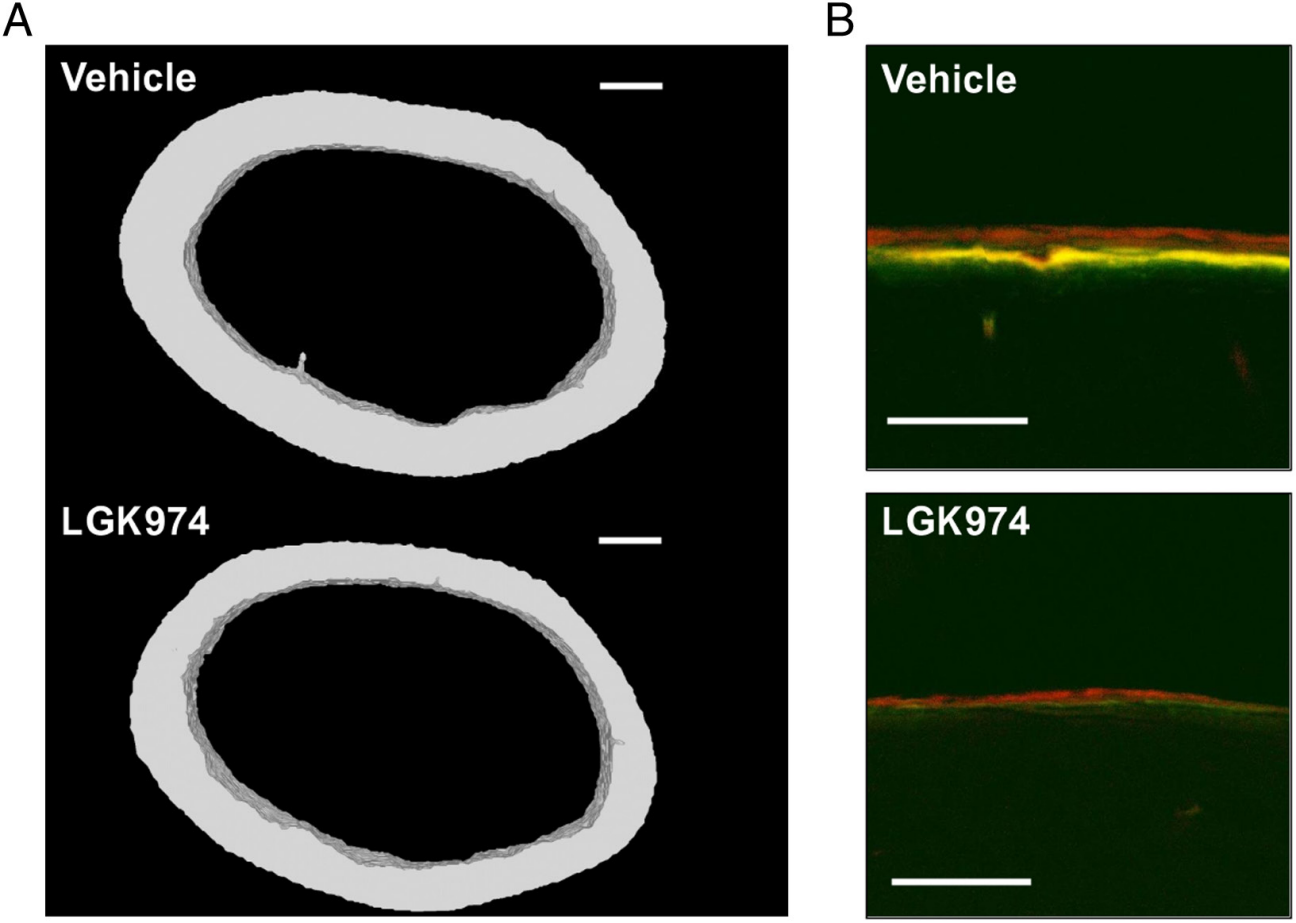
Cortical bone effects of LGK974. (A) 3D reconstruction of 50 axial sections from the femur midshaft of a representative animal treated with vehicle or LKG974 at 6 mg/kg/day (Bar: 200 µm). (B) Confocal microscopy images of the periosteal surface of the femur midshaft showing double labeling by calcein (green) 8 days before necropsy and by alizarin (red) 1 day before necropsy as a marker of new bone formation (Bar: 100 µm).

We then analyzed cortical bone structure and strength at necropsy. Both Porcupine inhibitors reduced cortical thickness and area of the femur midshaft assessed by µCT and the magnitude of the inhibitory effects of low dose and high doses of LGK974 were similar (all treatments *P* < 0.001, Fig. 3A, Table 1 and Supplementary Table 1, see section on supplementary data given at the end of this article). Representative 3D reconstructions of the femur midshaft of LGK974 and vehicle-treated mice are shown in Fig. 4A. Three-point bending test, evaluating the mechanical strength of the cortical bone in the diaphyseal region of tibia, revealed that all treatments with Porcupine inhibitors reduced the maximal failure load (low dose* LGK974*, *P* = 0.023; high dose* LGK974*, *P* < 0.001; *Wnt-C59*, *P* = 0.011; Fig. 3B and Supplementary Table 2).

**Table 1:** Microarchitecture of the cortical bone at the midshaft femur.

	Vehicle			Low-dose LGK974				High-dose LGK974				Wnt-C59			
	Mean	Lower 95% CI	Upper 95% CI	Mean	Lower 95% CI	Upper 95% CI	*P*	Mean	Lower 95% CI	Upper 95% CI	*P*	Mean	Lower 95% CI	Upper 95% CI	*P*
Cortical area (mm^2^)	**0.71**	0.69	0.72	**0.59**	0.57	0.61	**<0.001**	**0.56**	0.55	0.58	**<0.001**	**0.65**	0.62	0.67	**<0.001**
Polar moment of inertia (mm^4^)	**0.31**	0.30	0.32	**0.26**	0.25	0.27	**<0.001**	**0.24**	0.23	0.25	**<0.001**	**0.28**	0.26	0.30	**0.004**
Periosteal perimeter (mm)	**4.57**	4.53	4.61	**4.50**	4.46	4.55	0.075	**4.42**	4.39	4.46	**<0.001**	**4.53**	4.46	4.61	0.440
Endocortical perimeter (mm)	**3.47**	3.43	3.51	**3.58**	3.50	3.66	**0.016**	**3.54**	3.49	3.58	0.198	**3.53**	3.46	3.60	0.313

Microarchitecture parameters of cortical bone at the midshaft femur analyzed by µCT. Results represent the mean of the *n* = 10 animals per treatment group with 95% CIs and *P* values.

The mechanistic studies of the effect of Porcupine inhibition on cortical bone mass were performed using static and dynamic histomorphometry of femurs from mice treated with high-dose LGK974 or vehicle. LGK974 treatment reduced periosteal bone formation rate (Vehicle, 205 µm^3^/µm^2^/year, 95% CI 160–250; LGK974, 115 µm^3^/µm^2^/year, 95% CI 68–163, respectively; *P* = 0.006, Figs 3C, 4B and Supplementary Table 3) as a result of lower periosteal mineral apposition rate (*P* = 0.003) but unchanged mineralizing surface (*P* = 0.287; Fig. 3C) compared with vehicle treatment. There was no evidence of increased periosteal bone resorption assessed by the number of osteoclasts per bone perimeter (*P* = 0.085; Fig. 3C). At the endocortical surface (Fig. 3D), Porcupine inhibition increased the osteoclast number per bone perimeter (Vehicle, 2.9/mm, 95% CI 1.8–4.0; LGK974, 5.3/mm, 95% CI 3.6–6.9; *P* = 0.015) while bone formation rate was unaffected (*P* = 0.881; Fig. 3D).

The reduced periosteal bone formation and increased endocortical bone resorption were supported by the observation that *Alpl* mRNA levels (*P* = 0.032) were reduced while *Ctsk* mRNA levels (*P* < 0.001) and the *Rankl/Opg* ratio (*P* = 0.019) were increased in cortical bone by Porcupine inhibition (Fig. 3E).

### Trabecular bone impairment by Porcupine inhibitors

**Figure 5:**
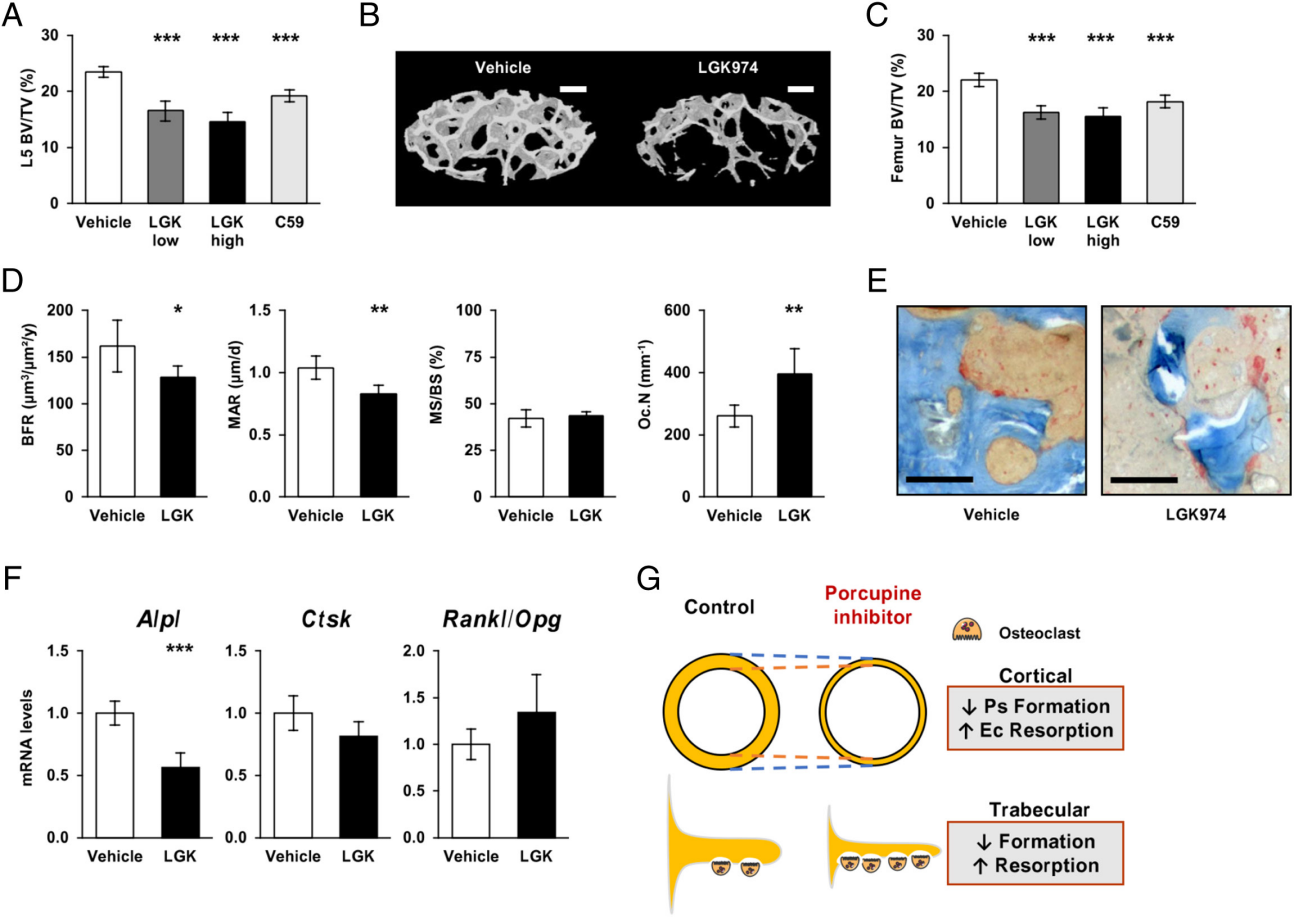
Effects of Porcupine inhibition on trabecular bone. (A) Trabecular bone volume fraction (BV/TV) in L5 vertebral body assessed by µCT. (B) 3D reconstruction of 200 axial sections from an elliptic region of interest within trabecular bone of the L5 vertebra body from a representative animal treated with vehicle or LKG974 at 6 mg/kg/day (Bar: 200 µm). (C) BV/TV in the distal metaphyseal region of the femur assessed by µCT. (D) Bone histomorphometry at the trabecular bone of L5 vertebral body showing bone formation rate (BFR), mineralization apposition rate (MAR), mineralising surface over bone surface (MS/BS) and osteoclast number per bone surface (Oc.N). (E) Microscopy images of the trabecular bone from L5 vertebra body stained with TRAP showing multinucleated osteoclasts at the bone surface (Bar: 50 µm). (F) *Alpl*, *Ctsk* and* Rankl/Opg ratio* mRNA levels in trabecular bone. (G) Summary diagram of the effects of Porcupine inhibition on cortical or trabecular bone formation and resorption. For each graph, *n* = 10 animals per group. Bars represent the mean and error bars represent the 95% CI of the mean. *P* values are indicated as *P < 0.05; **P < 0.01; ***P < 0.001.* Ps*, periosteal; *Ec*, endocortical.

Both Porcupine inhibitors reduced trabecular bone volume fraction (BV/TV) of the L5 vertebra body (all treatments *P* < 0.001, Fig. 5A, B and Supplementary Table 4) and the distal femur metaphysis (all treatments *P* < 0.001, Fig. 5C). These inhibitory effects on trabecular bone volume fraction were mainly caused by reduced number of trabeculae (*P* < 0.001; Table 2). High dose of LGK974 also reduced trabecular thickness at the L5 vertebra body (*P* = 0.001).

**Table 2:** Microarchitecture of the trabecular bone.

	Vehicle			Low-dose LGK974				High-dose LGK974				Wnt-C59			
	Mean	Lower 95% CI	Upper 95% CI	Mean	Lower 95% CI	Upper 95% CI	*P*	Mean	Lower 95% CI	Upper 95% CI	*P*	Mean	Lower 95% CI	Upper 95% CI	*P*
Metaphyseal distal femur															
Trabecular thickness (µm)	**48**	46	50	**46**	44	48	0.247	**46**	44	49	0.468	**47**	46	48	0.888
Trabecular number (/mm)	**4.6**	4.3	4.9	**3.6**	3.3	3.8	**<0.001**	**3.4**	3.1	3.6	**<0.001**	**3.9**	3.6	4.1	**<0.001**
Trabecular separation (µm)	**118**	116	120	**123**	121	125	**0.002**	**123**	122	125	**<0.001**	**122**	120	124	**0.006**
L5 vertebral body															
Trabecular thickness (µm)	**43**	42	45	**40**	37	43	0.083	**38**	35	41	**0.001**	**42**	40	44	0.505
Trabecular number (/mm)	**5.4**	5.2	5.7	**4.1**	3.7	4.5	**<0.001**	**3.8**	3.4	4.2	**<0.001**	**4.6**	4.4	4.8	**<0.001**
Trabecular separation (µm)	**137**	131	142	**160**	151	169	**<0.001**	**165**	158	173	**<0.001**	**153**	146	159	**0.004**

Microarchitecture parameters of trabecular bone at the metaphyseal distal femur and L5 vertebral body analyzed by µCT. Results represent the mean of the *n* = 10 animals per treatment group with 95% CI and *P* value.

The mechanistic studies of the effect of Porcupine inhibition on trabecular bone were performed using static and dynamic histomorphometry in the L5 vertebra body of high-dose LGK974 and vehicle-treated mice (Fig. 5D and Supplementary Table 3). Porcupine inhibition reduced trabecular bone formation rate (Vehicle, 162 µm^3^/µm^2^/year, 95% CI 128–195; LGK974, 128 µm^3^/µm^2^/year, 95% CI 113–143, respectively; *P* = 0.041) due to decreased mineral apposition rate (Vehicle, 1.0 µm/day, 95% CI 0.9–1.2; LGK974, 0.8 µm/day, 95% CI 0.8–0.9, respectively; *P* = 0.003) but unaffected mineralizing surface (*P* = 0.588). LGK974 increased the number of osteoclasts per trabecular bone perimeter (Vehicle, 260/mm, 95% CI 218–302; LGK974, 396/mm, 95% CI 299–492, respectively; *P* = 0.009, Fig. 5D and E).

The reduced trabecular bone formation was supported by reduced *Alpl* mRNA levels in the vertebral body enriched in trabecular bone (*P* < 0.001; Fig. 5F). However, *Ctsk* mRNA levels (*P* = 0.057) and *Rankl/Opg* ratio (*P* = 0.146) were unchanged in trabecular bone (Fig. 5F). *Opg* mRNA levels alone was however significantly decreased (Vehicle, 1.00, 95% CI 0.83–1.17; LGK974, 0.77, 95% CI 0.61–0.93, respectively; *P* = 0.040).

## Discussion

Porcupine inhibitors are under development for cancer therapy, but their possible side effects on bone health are unknown. We, herein, demonstrate that Porcupine inhibitors severely impair bone mass and strength and that this impairment is caused by a combination of reduced bone formation and increased bone resorption (Fig. 5G).

Although Porcupine expression was higher in cortical diaphyseal bone than in the vertebral body, our expression analyses demonstrate that Porcupine is expressed in most tissues and cells expressing WNTs including both osteoblasts and osteoclasts. In addition, the expression was mainly unaffected during osteoblast and osteoclast differentiation, indicating that Porcupine expression is only marginally regulated.

We evaluated the skeletal effects of two Porcupine inhibitors and observed that they both reduced total body BMD, cortical bone thickness and trabecular bone volume fraction, strongly suggesting that these deleterious effects on bone mass is a class effect of Porcupine inhibitors. Furthermore, both inhibitors reduced the mechanical strength of the bones when evaluated using three-point bending test, suggesting that potential cancer therapies using Porcupine inhibitors may result in increased fracture risk. Skeletal side effects of other novel cancer therapies targeting the WNT pathway have recently been described (Smith *et al.* 2013, O’Cearbhaill *et al.* 2016). Among them, vantictumab (Smith *et al.* 2013), a monoclonal antibody against several Frizzled (FZD) receptors and ipafricept (O’Cearbhaill *et al.* 2016), a decoy receptor for WNT ligands, were shown to exert severe effects on bone remodeling, and a case of drug-related fracture was reported.

Our mechanistic studies revealed that Porcupine inhibitors reduced cortical bone thickness via a combination of reduced periosteal bone formation rate and increased endocortical bone resorption (Fig. 5G). The reduced periosteal bone formation was the result of reduced mineral apposition rate, indicating reduced osteoblast activity, while mineralized bone surface was unaffected. A limitation with our study is that we did not count the number of osteoblasts or measured the osteoid thickness. Therefore, we cannot exclude that the osteoblast numbers were also affected. The increased endocortical bone resorption was supported by increased number of osteoclasts on the endocortical bone surface as well as increased *Ctsk* expression and increased *Rankl/Opg* ratio in the cortical bone. Both Porcupine inhibitors reduced trabecular bone volume fraction in both the axial and the appendicular skeleton, mainly via a reduced number of bone trabeculae. The loss of trabeculae was caused by decreased trabecular bone formation and increased number of osteoclasts (Fig. 5G). Porcupine inhibition reduced *Opg* expression in the present study, and it was previously shown that canonical WNT signaling regulates *Opg* expression by osteoblasts and thereby controls osteoclast differentiation (Glass *et al.* 2005). In addition, PINP, a marker of bone formation, was reduced while CTX, a marker of bone resorption, was increased in serum, further supporting an imbalance of bone formation and resorption. The effects of Porcupine inhibition on both bone formation and bone resorption are in line with previous studies describing that WNT signaling exerts major effects not only on bone formation but also on bone resorption (Baron & Hesse 2012, Lerner & Ohlsson 2015).

The severe bone loss by Porcupine inhibition, affecting both cortical and trabecular bone mass, is similar to the findings from studies targeting Wls in mice. Osteoblast-specific Wls deleted mice display reduced bone formation and increased resorption leading to dramatic trabecular and cortical bone loss and spontaneous fractures (Zhong *et al.* 2012). Previous studies have demonstrated that certain WNTs exert bone compartment-specific effects. We reported that osteoblast-derived WNT16 increases cortical but not trabecular bone mass (Moverare-Skrtic *et al.* 2014) while others have shown that *WNT10b^−^^/^^−^* mice develop an age-dependent loss of trabecular bone specifically (Stevens *et al.* 2010). Therefore, it is not surprising that both mice with Wls inactivation and mice treated with Porcupine inhibitors, targeting all WNTs expressed, have a severe skeletal phenotype affecting both trabecular and cortical bone mass. Interestingly, the activation of the WNT canonical pathway by romosozumab induces opposite effects of bone turnover markers, with a transient increase in bone formation marker and sustained decrease in bone resorption marker (McClung *et al.* 2014).

Since Porcupine inhibition both reduces bone formation and increases bone resorption, the concomitant use of anti-resorbing agents with Porcupine inhibitors could blunt the drug-induced bone loss. Moreover, in the situation of cancer, osteoclasts contribute to the development of bone metastasis and by releasing growth factors out of the resorbed bone, osteoclasts may also promote tumor growth, leading to a vicious circle (Roodman 2004). In addition to their bone-sparing effects, bisphosphonates or denosumab treatments also impede metastases, complementing the anti-tumor effects of Porcupine inhibitors (Coleman *et al.* 2012).

In conclusion, Porcupine inhibitors exert deleterious effects on bone mass and strength caused by a combination of reduced bone formation and increased bone resorption. We speculate that possible new cancer treatments using Porcupine inhibitors may increase the risk of fractures.

## Supplementary data

Supplemental Table 1

Supplemental Table 2

Supplemental Table 3

Supplemental Table 4

## Declaration of interest

The authors declare that there is no conflict of interest that could be perceived as prejudicing the impartiality of the research reported.

## Funding

This work was supported by funding from the European Union’s Horizon 2020 Marie Skłodowska-Curie Actions research and innovation programme under grant agreement GOTBONE-750579, the French Society of Rheumatology, Vinnova, the Swedish Research Council, the Swedish Foundation for Strategic Research, the ALF/LUA research grant in Gothenburg, the Lundberg Foundation, the Knut and Alice Wallenberg Foundation, the Torsten Söderberg Foundation, and the Novo Nordisk Foundation.

## Author contribution statement

Study design: T F B, S M S and C O. Study conduct: T F B, K N and R B. Data collection: T F B, K N, S M S and P H. Data analysis: T F B, P H, A K, J T and M C S. Data interpretation: T F B, R B, P H, J T, M C S, U L, S M S and C O. Drafting manuscript: T F B and S M S. Revising manuscript content: U L and C O. Approving final version of manuscript: T F B, K N, R B, P H, U L, A K, J Y, M C S, S M S and C O. T F B and C O take responsibility for the integrity of the data analysis.
